# Surface Plasmon Resonance Analysis Shows an IgG-Isotype-Specific Defect in ABO Blood Group Antibody Formation in Patients with Common Variable Immunodeficiency

**DOI:** 10.3389/fimmu.2015.00211

**Published:** 2015-05-06

**Authors:** Michael B. Fischer, Wendelin Wolfram, Christoph J. Binder, Georg A. Böhmig, Markus Wahrmann, Martha M. Eibl, Hermann M. Wolf

**Affiliations:** ^1^Department of Transfusion Medicine, Medical University of Vienna, Vienna, Austria; ^2^Center of Biomedical Technology, Danube University Krems, Krems an der Donau, Austria; ^3^Department of Laboratory Medicine, Medical University of Vienna, Vienna, Austria; ^4^Division of Nephrology and Dialysis, Department of Medicine III, Medical University of Vienna, Vienna, Austria; ^5^Immunology Outpatient Clinic, Vienna, Austria

**Keywords:** blood groups, isoagglutinins, common variable immunodeficiency, B cell subsets, anti-ABO antibodies, pneumococcal polysaccharide, natural antibodies

## Abstract

**Background:**

Common variable immunodeficiency (CVID) is the most common clinically severe primary immunodeficiency and comprises a heterogeneous group of patients with recurrent severe bacterial infections due to the failure to produce IgG antibodies after exposure to infectious agents and immunization. Diagnostic recommendations for antibody failure include assessment of isoagglutinins. We have readdressed this four decades old but still accepted recommendation with up to date methodology.

**Methods:**

Anti-A/B IgM- and IgG-antibodies were measured by Diamed-ID Micro Typing, surface plasmon resonance (SPR) using the Biacore^®^ device and flow cytometry.

**Results:**

When Diamed-ID Micro Typing was used, CVID patients (*n* = 34) showed IgG- and IgM-isoagglutinins that were comparable to healthy volunteers (*n* = 28), while all XLA patients (*n* = 8) had none. Anti-A/B IgM-antibodies were present in more than 2/3 of the CVID patients and showed binding kinetics comparable to anti-A/B IgM-antibodies from healthy individuals. A correlation could be found in CVID patients between levels of anti-A/B IgM-antibodies and levels of serum IgM and PnP–IgM-antibodies. In contrast in CVID patients as a group ABO antibodies were significantly decreased when assessed by SPR, which correlated with levels of switched memory, non-switched memory and naïve B cells, but all CVID patients had low/undetectable anti-A/B IgG-antibodies.

**Conclusion:**

These results indicate that conventional isoagglutinin assessment and assessment of anti-A/B IgM antibodies are not suited for the diagnosis of impaired antibody production in CVID. Examination of anti-A/B IgG antibodies by SPR provides a useful method for the diagnosis of IgG antibody failure in all CVID patients studied, thus indicating an important additional rationale to start immunoglobulin replacement therapy early in these patients, before post-infectious sequelae develop.

## Introduction

Common variable immunodeficiency (CVID) is a heterogeneous disorder belonging to the group of primary antibody deficiencies, characterized by markedly reduced serum levels of IgG, IgA, and often IgM, and a substantial failure to produce specific IgG antibodies after vaccination or exposure to foreign antigens (1–5). This renders patients susceptible to recurrent sinopulmonary, ear, and gastrointestinal infections, the underlying regulatory immune defect can lead to autoimmune diseases, and certain malignancies may occur more frequently. Several monogenetic defects including homo/heterozygous mutations in ICOS (inducible costimulator of activated T cells), TACI (transmembrane activator and calcium-modulator and cyclophilin ligand interactor), BAFF-R (B cell activating factor receptor), CD19, CD81, and Msh5 were found in CVID patients, but genetics of CVID are complicated by unaffected relatives that carry the same mutations showing that the genotype–phenotype correlation can be variable (6). For the majority of CVID patients, however, the underlying molecular defect has not yet been defined. Immunological abnormalities including defects in antigen presentation by monocytic cells and perturbed differentiation, maturation, and function of DCs and invariant NK (iNK) cells were described (7, 8). In addition to intrinsic B cell defects (9, 10) functional abnormalities in T cells which can potentially lead to defects in memory B cell compartment were described, and included impaired early signaling events, production of cytokines and expression of activation markers after antigenic stimulation (11, 12).

Early IUIS, ESID/PAGID, and recent ESID diagnostic criteria of CVID include poor antibody response to polypeptide and polysaccharide vaccines and absence of anti-blood group A (anti-A) and B (anti-B) antibodies as equivalent criteria (3–5). However, studies addressing the question whether CVID patients have defective production of isoagglutinin antibodies examined in an isotype-specific manner are scarce. The immunologically active epitopes of blood group ABO antigens share carbohydrate moieties (13). Blood group A/B specific antibodies are produced by all individuals with a functional immune system and are included under the broad classification of ‘natural antibodies’ in humans (14). They appear first in infants at the age of 3–6 month when the developing immune system can react with microorganisms and environmental antigens that have carbohydrate epitopes similar to the blood group ABO antigens (13, 14). The aim of the present study was to investigate whether freshly diagnosed CVID patients, who hitherto had not received IVIG treatment, show a defect in anti-A/B IgG or IgM antibodies. We used three different techniques, the DiaMed-ID Micro Typing System, a blood group ABO flow cytometry based assay (15), and the surface plasmon resonance technology (SPR) using the Biacore^®^ device and synthetic AB trisaccharides (16–18) to study the blood group anti-A/B IgG and IgM antibody titer in an isotype-specific assay and the on/off rates of the binding of the respective antibodies to defined blood group A/B trisaccharides in CVID patients and healthy individuals.

## Materials and Methods

### CVID patients and healthy controls

Thirty-four patients with CVID diagnosed according to the criteria established by the IUIS expert committee (5) were included in the study after informed consent was obtained. The immunological characterization of the patients is depicted in Table 1. The study was conducted according to guidelines of the Medical University of Vienna (MUW) and was approved by the ethic commission of the MUW. The patients gave their informed consent that anonymized data collected as part of the routine medical attendance the patients received could be included in a scientific publication. All results presented in this study were obtained as part of the routine medical attendance the patients received, no extra venipuncture was performed on the basis of this study. Serum samples of CVID patients were drawn before regular intravenous IVIG substitution therapy was started. Twenty-eight healthy adult blood donors and eight X-linked primary antibody deficiency (XLA) patients with no B cells and complete absence of immunoglobulins of all isotypes due of a defect in bruton’s tyrosine kinase (btk) served as positive and negative controls (individuals with blood group AB were not included in the study). The distribution of ABO blood group among the controls (blood group [N]: O [16], A [7], B [5], AB [0]) was not significantly different as compared to the CVID patients (Table 1; *P* = 0.1, Chi-squared analysis). All serum samples were immediately separated from peripheral blood, dispensed into 500 μl aliquots and stored until analysis at –30°C. IgG from plasma of eight healthy subjects with known history of high anti-A/B antibodies was depleted using a protein A column first and subsequently a Protein G column (Enchant™ IgG purification and depletion Kit, Pall Life Science, Port Washington, NY, USA) in order to obtain IgM antibodies only. The amount of IgM as well as IgG contamination was determined by ELISA.

**Table 1:** **Immunological characterization of CVID patients**.

Patient number	Sex	Age^a^	ABO blood group	Serum immunoglobulins (mg/dl)^a^			PnP antibodies (reciprocal titer)^b^		CD19 + (% ly)^c^	% of CD19-positive lymphocytes		
				IgG	IgA	IgM	PnP–IgG	PnP–IgM		CD27+ IgD−	CD27+ IgD+	CD27− IgD+
1	m	29	A	223	6	5	<20	<20	6	1,48	5,33	84,68
2	m	40	A	86	<6	12	<20	<20	6	4,75	6,27	83,17
3	m	47	A	160	6	4	21	<20	7	4,67	20,46	70,29
4	f	36	O	48	<7	<6	<20	<20	8	0,38	0,98	94,03
5	f	52	n.a.	<7	<6	12	<20	<20	4	n.a.	n.a.	n.a.
6	f	48	n.a.	12	6	8	<20	<20	14	1,60	29,73	66,40
7	m	27	B	22	<6	<5	<20	<20	0	n.a.	n.a.	n.a.
8	f	49	O	94	6	23	<20	<20	5	0,66	4,80	91,95
9	f	29	O	169	<8	<7	63	<20	15	n.a.	n.a.	n.a.
10	f	54	O	199	<8	49	63	35	18	1,63	16,86	79,52
11	f	34	B	239	<8	47	<20	40	12	2,49	11,77	84,42
12	m	39	A	111	<7	<6	n.a.	n.a.	9	n.a.	n.a.	n.a.
13	m	10	O	365	<5	17	<20	20	14	1,94	9,28	85,78
14	m	39	O	288	7	6	99	<20	13	1,60	41,62	49,28
15	m	50	O	<158	59	171	<20	49	18	n.a.	n.a.	n.a.
16	f	30	O	179	36	252	39	121	8	0,68	48,39	49,26
17	f	57	A	204	11	34	<20	30	20	14,52	17,86	62,60
18	m	33	A	84	<7	9	<20	<20	20	0,39	1,68	96,69
19	m	64	A	89	11	11	<20	36	3	n.a.	n.a.	n.a.
20	m	32	A	9	6	4	<20	<20	13	n.a.	n.a.	n.a.
21	f	44	A	219	44	26	<20	<20	5	n.a.	n.a.	n.a.
22	f	57	A	197	<8	<7	n.a.	n.a.	6	n.a.	n.a.	n.a.
23	m	20	A	<148	<8	<6	<20	<20	5	1,80	6,10	87,02
24	m	42	O	60	<7	<4	<20	<20	15	8,91	29,57	59,65
25	m	13	A	76	<8	50	<20	<20	14	n.a.	n.a.	n.a.
26	m	27	A	<148	<8	41	39	<20	11	1,98	28,37	62,33
27	m	25	O	10	<6	8	<20	<20	13	0,77	25,32	73,26
28	f	10	O	383	356	76	<20	<20	5	n.a.	n.a.	n.a.
29	m	50	A	<7	<6	<5	<20	<20	3	2,75	9,89	84,07
30	m	19	O	210	<6	39	<20	<20	12	0,93	4,34	91,09
31	f	21	O	295	46	30	<20	117	10	1,35	7,72	89,37
32	m	22	A	154	13	31	n.a.	n.a.	10	1,83	14,05	83,28
33	f	47	A	201	9	33	<20	50	13	1,90	15,81	80,98
34	m	17	O	<7	<6	<5	<20	<20	0	n.a.	n.a.	n.a.
Normal range				815–1784	93–287	108–237	>200	>100	7–23	9–36	6–32	37–77

*^a^At time of diagnosis, before the initiation of immunoglobulin replacement therapy*.

*^b^Serum IgG- and IgM-antibodies against 23-valent pneumococcal polysaccharides (PnP) were determined by ELISA before the initiation of immunoglobulin replacement therapy*.

*^c^Percentage of peripheral blood CD19-positive lymphocytes as determined by flow cytometry*.

*n.a., Data not available*.

### Determination of anti-A/B antibodies by the DiaMed-ID micro typing system

Anti-A/B antibodies were determined by the Diamed-ID Micro Typing System (DiaMed AG, Cressier, Switzerland) according to the manufacturer’s protocol. The ID-Card 50520 (NaCl) was used to measure IgM, while the ID-Card 50531 (LISS/Coombs containing polyspecific anti-human gammaglobulin [AHG] serum) could determine IgG in addition to IgM. The serum samples were serially diluted with 0.9% saline solution starting at 1:2, 1:4, until 1:265, and 25 μl of each dilution was pipetted into a single column of the microtube gel card together with 50 μl of DiaMed ID-DiaCell ABO/I–II test cell suspensions A_1_ or B (DiaMed AG). The ID-Cards (NaCl) were subsequently incubated at room temperature for 15 min, the ID-Cards (LISS/Coombs) at 37°C and then centrifuged in a DiaMed ID-Centrifuge 24 s for 10 min at 910 rpm. Results were read immediately after centrifugation, the titer that led to visible agglutination of erythrocytes dispersed in the gel was selected positive. If an individual showed both anti-A and anti-B antibodies then the higher titer of the two values was used for statistical analysis.

### Flow cytometric analysis of patients’ anti-A/B antibodies and B cell populations

Undiluted serum samples of CVID patients and healthy control subjects were incubated for 30 min at 4°C with 1% solution of fixed RBCs (15). Thereafter, cells were washed two times in FACS buffer (Hank’s buffered salt solution with 0.1% BSA) and bound anti-A/B antibody to test erythrocytes was measured by flow cytometry using either a DyLight 649 conjugated affinity purified Fab fragment goat anti-human IgM (Fc_5μ_ fragment specific, 15 μg/ml, Jackson ImmunoResearch Ltd, Newmarket, UK) or a DyLight 649 conjugated affinity purified F(ab′)_2_ fragment rabbit anti-human IgG (Fc_γ_ fragment specific, 60 μg/ml, Jackson ImmunoResearch). A minimum of 10, 000 events was acquired. All analysis were performed on a BD FACSCanto™(Becton Dickinson, Franklin Lakes, NJ, USA) interfaced to BD FACSDiva™ software. Mean fluorescence intensity (MFI) was calculated and represented the titer of anti-A/B IgG or IgM antibodies present in the sample. If an individual sample showed both anti-A and anti-B IgG and IgM antibodies then the higher MFI-titer of the two values was used for statistical analysis. Lymphocyte subpopulations and B cell subsets were analyzed in peripheral whole blood by flow cytometry using standard protocols with commercially available directly conjugated monoclonal antibodies (anti-CD19 PerCP, Becton Dickinson Austria Ges.m.b.H., Schwechat, Austria; anti-IgD FITC, Becton Dickinson Austria Ges.m.b.H., anti-CD27 PE, eBioscience, Vienna, Austria) and a FACSCalibur (Becton Dickinson Austria Ges.m.b.H.). Data analysis was performed using CellQuest software (Becton Dickinson Austria Ges.m.b.H.).

### Determination of anti-A/B antibodies by surface plasmon resonance

The erythrocyte aggregation assay based on the Diamed-ID Micro Typing System as well as the flow cytometric method to quantify binding of anti-A/B IgG or IgM antibodies to fixed human A or B red blood cells (ABO-FACS) are semi-quantitative assays that lack sensitivity and specificity to precisely rank anti-A/B IgG and IgM antibodies according to their strength of reaction with the corresponding blood group A/B trisaccharide antigens. Therefore, we used SPR to measure the association rate (*k*_a_) of the anti-A/B IgG and IgM antibodies to the immobilized synthetic blood group A or B trisaccharides and their dissociation rate (*k*_d_) on a Biacore X device (16–18). We used the dextran hydrogel layer on the CM-5 sensor chip to form a hydrophilic environment for the attachment of blood group A or B trisaccharide amine derivative, preserving the blood group antigens in a non-denatured state (18). This allows for measurement of antigen–antibody binding under almost physiological conditions. Amine-labeled blood group A and B trisaccharides (Dextra Laboratories Ltd., Reading, UK) were immobilized on CM5 sensor chips (Biacore, GE Healthcare) according to standard protocols (16). In brief, the CM-5 sensor chip surface was activated with 100 μl of 0.05 mol/l *N*-hydroxysuccinimide (Sigma Aldrich, St. Louis, MO, USA) and 0.2 mol/l *N*-ethyl-*N*’-dimethylamino propylcarbodiimide (Sigma Aldrich) injected into the buffer stream of HBS-EP (0.01 mol/l HEPES buffer, pH 7.4, containing 0.15 mol/l NaCl, 3 mmol/l EDTA, and 0.005% surfactant P20). The buffer stream was passed through FC-1 and FC-2 of the Biacore X instrument (GE Health Care, Uppsala, Sweden) at a flow rate of 5 μl/min for 20 min. Subsequently, 15 μl of a blood group A/B trisaccharide amine derivative solution at a concentration of 1 mg/ml, prepared with borate (pH 8.5), was injected into the buffer stream and passed through FC-1 at a flow rate of 5 μl/min and was halted after injection of 7 μl for 2 h. Residual active ester groups on the sensor surface were then deactivated by injecting 100 μl of 1 mol/l ethanolamine-HCl (pH 8.5) into the buffer stream and passing it through FC-1 and FC-2, and by passing the buffer stream through the cell until a stable baseline was achieved. To measure anti-A/B antibodies, crude human serum was diluted by half with the HBS-EP and 100 μl of the diluted plasma samples were passed through FC-1 and FC-2 at a flow rate of 20 μl/min for 5 min. When the association phase was finished, the flow cell was washed for 20 s with the HBS-EP buffer and dissociation was recorded in resonance units (RU) at a flow rate of 20 μl/min for 20 min. The chip was regenerated after each measurement in order to reach the same baseline level as prior to measurement by removing bound antibody with 50 μl of 50 mmol/l NaOH buffer at a flow rate of 60 μl/min. To establish a calibration curve for anti-A/B antibody concentration, BIAevaluation software (General Electric Healthcare) was used. The amount of anti-A or anti-B antibody that associated with the corresponding blood group A or B trisaccharide immobilized on the sensor chip surface was obtained by subtracting the FC-II value (RU) from the FC-I value (RU). If an individual showed both anti-A and anti-B antibodies then the higher of the two RU values was used for statistical analysis.

### Isotype-specific determination of anti-A/B antibodies by surface plasmon resonance

To measure anti-A/B IgG and IgM antibodies, crude human serum was diluted by half with the HBS-EP and 100 μl of the diluted plasma samples were passed through FC-1 and FC-2 at a flow rate of 20 μl/min for 5 min. To discriminate between anti-A/B IgM and IgG response, anti-human IgG Abs (Coombs serum, Diamed) were injected first after the association phase was finished, followed by injection of anti-human IgM mAbs (Sigma Aldrich). The increase in RU following the injection was indicative for the amount of IgG or IgM bound to the synthetic trisaccharides. If an individual showed both anti-A and anti-B IgG or IgM antibodies then the specificity with the higher titer (anti-A or anti-B) of the respective immunoglobulin isotype was used for statistical analysis. Before analysis was started, the binding of commercially available anti-blood group A/B antisera (anti-A murine monoclonal Abs clone MH04 and A3D3, anti-B murine monoclonal Abs clone NB1.19, NB10.5A5, and NB10.3B4, Ortho-Clinical Diagnostics, Neckargmünd, Germany) to the respective trisaccharides was measured in order to examine chip performance. Serial dilutions of anti-blood group A mAbs (IgG clone 9A, Abcam, Cambridge, MA, USA) and anti-blood group A/B antisera (Ortho-Clinical Diagnostics) were performed to generate a dose-dependent curve.

### Measurement of serum immunoglobulins and serum antibodies against pneumococcal polysaccharides

Serum concentrations of IgG, IgA, and IgM were determined by laser nephelometry using reagents purchased from Siemens–Behring Division (Siemens Healthcare Diagnostics GmbH, Vienna, Austria). Serum levels of IgG- and IgM-antibodies against 23-valent pneumococcal capsular polysaccharide were determined using an in-house produced ELISA detecting all serotypes contained in the 23-valent PnP vaccine as previously described (19, 20).

### Statistical analysis

The non-parametric Mann–Whitney *U*-test (two-sided *P* value) was used for unpaired comparison of results obtained from patients and healthy control subjects. A *P* value of <0.05 was assumed to be statistically significant. The Bravais–Pearson-correlation coefficient was calculated to determine a statistically significant correlation between two parameters at a level of *P* < 0.05. Where results are depicted using box plot diagrams, the median of the study group is represented by a cross, the interquartile range (IQR) is represented by the box, 5- and 95-percentile values are represented by the whiskers, and minimum and maximum values are represented by circles. The 95% confidence interval for the median calculated according to McGill et al. (21) is indicated by horizontal bars.

## Results

### Blood group anti-A/B antibodies did not differ between CVID patients and healthy individuals when determined by diamed-ID Micro typing

**Figure 1:**
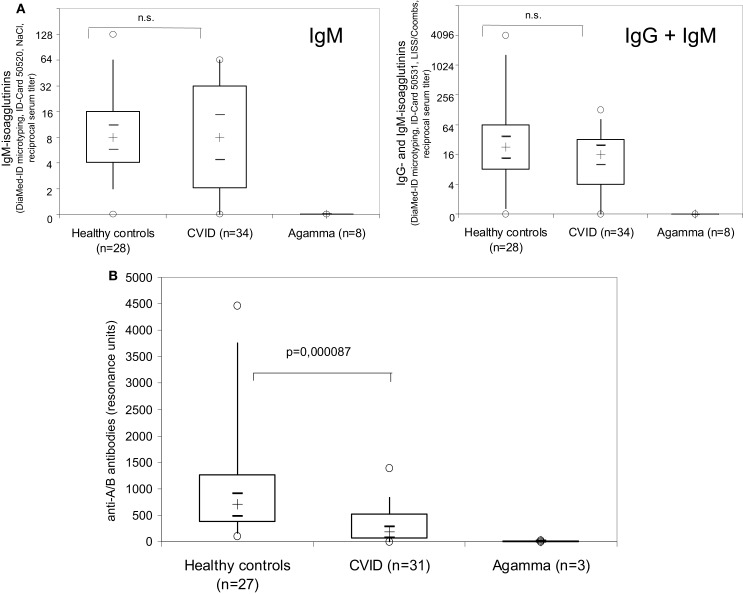
**Examination of anti-A/B antibodies in CVID patients, patients with XLA and healthy controls using the Diamed-ID Micro Typing System with the ID-Card 50520 (NaCl) to measure IgM isoagglutinins [(A), left panel] or with the ID-Card 50531 (LISS/Coombs containing polyspecific AHG serum) to determine IgG- in addition to IgM-isoagglutinins [(A), right panel] and surface plasmon resonance (SPR) technology without identification of the antibody isotype using synthetic blood group A/B trisaccharides coupled to the CM-5 Biacore^®^ chip by amine binding (B)**. Reciprocal serum isoagglutinin titers [**(A)**, left panel, IgM isoagglutinins; **(A)**, right panel, IgG and IgM isoagglutinins] and SPR resonance units **(B)** are depicted using box plot diagrams, the median of the study group is represented by a cross, the interquartile range (IQR) is represented by the box, 5- and 95-percentile values are represented by the whiskers, and minimum and maximum values are represented by circles. The 95% confidence interval for the median calculated according to McGill et al. (21) is indicated by horizontal bars. The non-parametric Mann–Whitney *U*-test (two-sided *P* value) was used for statistical comparison between patients and healthy controls.

When the amount of anti-A/B antibodies in samples of 34 CVID patients, diagnosed according to the criteria established by the IUIS expert committee (5), was determined by the Diamed-ID Micro Typing System using the ID-Card 50520 (NaCl) to measure IgM-isoagglutinins, no difference could be found between CVID patients and healthy individuals (Figure 1A, left panel). X-linked agammaglobulinemia (XLA) patients with a defect in btk showed no detectable isoagglutinin antibodies (Figure 1A, left panel). When samples were further analyzed in the ID-Card 50531 (LISS/Coombs containing polyspecific AHG serum) to determine IgG- in addition to IgM-isoagglutinins, the isoagglutinin titers detected were higher as compared to the NaCl-system, but still no difference was observed between CVID patients and controls (Figure 1A, right panel).

### Surface plasmon resonance analysis shows defective ABO blood group antibody formation in CVID patients

In the erythrocyte aggregation assay based on the Diamed-ID Micro Typing System, 4/28 (14%, NaCl system) and 3/28 (11%, LISS/Coombs system) of the healthy volunteers had low to undetectable (serum titer ≤ 1:2) isoagglutinin antibodies (data not shown). To better detect anti-A/B antibodies, SPR technology using synthetic blood group A/B trisaccharides coupled to the CM-5 sensor chip by amine binding was employed (16–18). This technology uses a molecularly defined carbohydrate antigen and allows for real-time analysis of molecular interactions of unlabeled antigen and antibody, thus leaving the functional structures of both interaction partners intact. Furthermore, the amine-labeling technology allows immobilization with a controlled orientation for the carbohydrates (18). Thus the sensitivity of detection of anti-A/B antibodies is enhanced by SPR technology (16). Accordingly, in those healthy individuals where no or very low detectable anti-A/B titer could be observed in the DiaMed-ID Micro Typing System and no binding of anti-A/B specific antibodies to erythrocytes could be detected in the flow cytometry based assay (data not shown), SPR showed slight but detectable binding (100–130 RU) of anti-A/B antibodies to blood group A/B tri-saccharides, and 27/27 (100%) of healthy individuals had anti-AB antibody titers above 100 RU (Figure 1B). In contrast to the isotype-specific SPR assay employed in later experiments, the isotype of the anti-A/B antibodies was not identified in these analyses.

**Figure 2:**
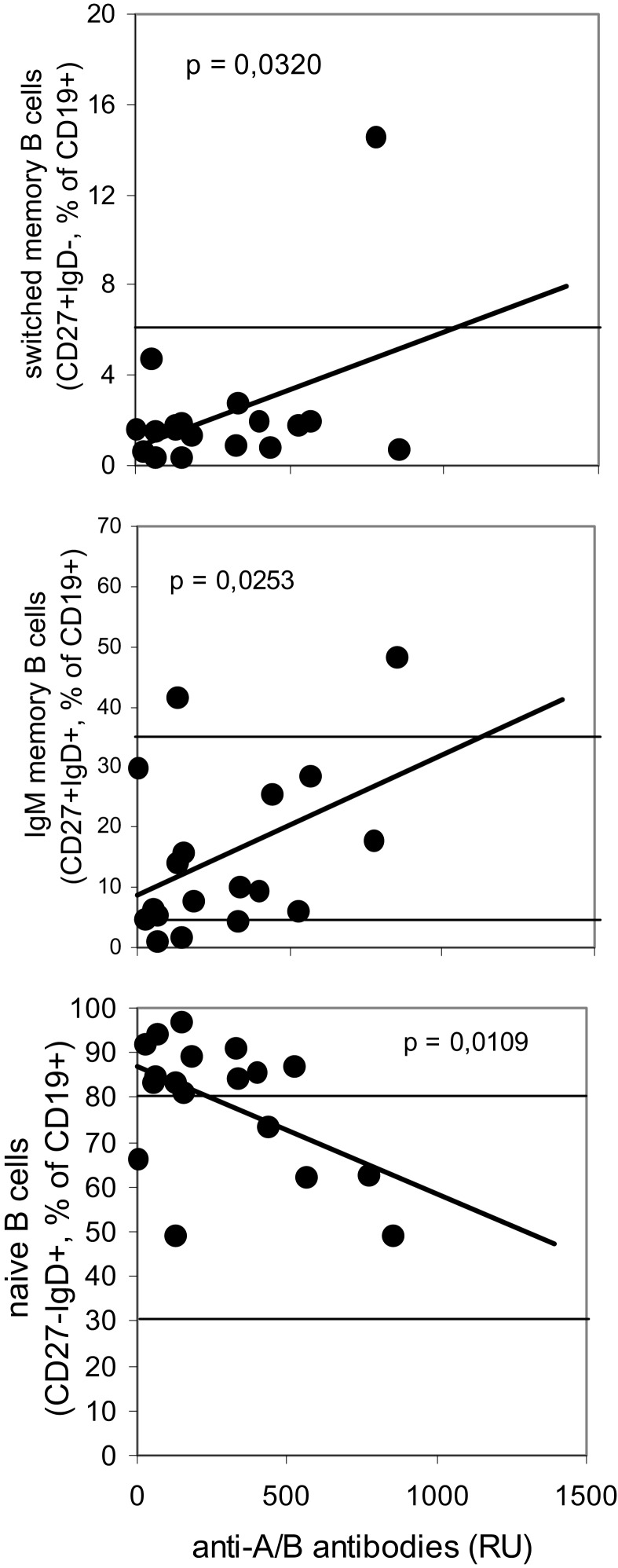
**Correlation between levels of anti-A/B antibodies as detected by surface plasmon resonance using the Biacore^®^ device and levels of B cell subsets in CVID patients**. Anti-A/B antibodies were determined using surface plasmon resonance (SPR) technology with synthetic blood group A/B trisaccharides coupled to the CM-5 Biacore^®^ chip by amine binding, antibody titers are expressed as resonance units (RU). Peripheral B cell subsets were determined by three-color-flow cytometry, results are depicted as the percentage of total number of CD19^+^ B lymphocytes. Horizontal lines indicate the normal range of B cell subsets as determined in 134 healthy individuals (upper panel: 5th percentile; middle and lower panel: upper line, 95th percentile, lower line, 5th percentile). The Bravais–Pearson-correlation coefficient was calculated to determine a statistically significant correlation between two parameters at a level of*P* < 0.05.

In contrast to the findings obtained using the Diamed-ID Micro Typing System, serum titers of anti-A/B antibodies as detected by SPR were significantly decreased in CVID patients as compared to healthy controls (Figure 1B). In 22/31 CVID patients (in whom both assays could be performed in parallel) anti-A/B antibodies were detectable in the erythrocyte aggregation assay, and these patients also showed antibody binding to the A/B trisaccharides immobilized on the surface of the chip as indicated by SPR-RU, although with a significantly decreased titer. The remaining nine patients showed no visible aggregation in the Diamed-ID Micro System and also showed no or only very low antibody binding to the trisaccharides (RU well below 100). There was a positive correlation between the patients’ decreased levels of anti-A/B antibodies as detected by SPR and decreased levels of switched memory B cells and IgM memory B cells, and patients with low anti-A/B IgM antibodies had higher levels of naïve B cells, thus showing that SPR anti-A/B antibodies are decreased in parallel with other parameters indicative of the defect in B cell function present in CVID patients (Figure 2).

### CVID patients produce no blood group anti-A/B IgG as examined by flow cytometry

**Figure 3:**
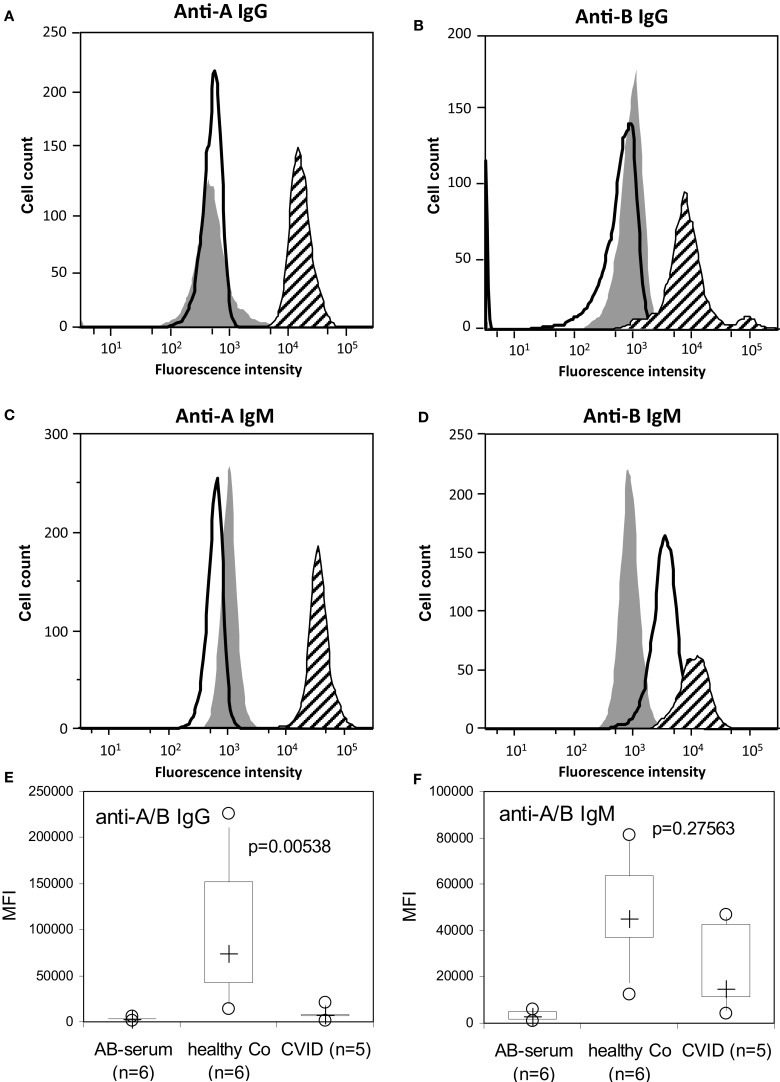
**Detection of anti-A/B IgG- and IgM-antibodies by flow cytometry**. The anti-A/B IgG and IgM mean fluorescence intensity (MFI) histograms of a representative CVID patient (bold lines) with no anti-A/B IgG **(A,B)**, no anti-A IgM but with anti-B IgM present **(C,D)**, and a healthy individual (hatched histograms) with anti-A/B IgG **(A,B)** and anti-A/B IgM present **(C,D)** are depicted. Background binding of fluorescence-labeled anti-IgG and anti-IgM was assessed using AB serum [**(A–D)**, filled histograms] containing no or only low levels of anti-A/B IgG-**(A,B)** and IgM-antibodies **(C,D)**. The *X*-axis represents the MFI given on a logarithmic scale and the *Y* -axis the cell number on a linear numeric scale. Defective anti-A/B IgG **(E)** but normal IgM **(F)** antibody response in patients with CVID and controls (healthy Co) as examined by flow cytometry. Background binding of fluorescence-labeled anti-IgG and anti-IgM to the erythrocytes was assessed in the presence of an AB serum containing no or only low levels of anti-A/B antibodies. Box plot diagrams show anti-A/B IgG **(E)** and IgM **(F)** antibodies in serum with the median of the study group represented by a cross, the interquartile range (IQR) represented by the box, 5- and 95-percentile values represented by the whiskers, and minimum and maximum values depicted by circles. The non-parametric Mann–Whitney *U*-test (two-sided *P* value) was used for statistical comparison between patients and healthy control subjects.

To further discriminate between blood group anti-A/B IgM- and IgG-antibodies, patients’ samples were analyzed by a semi-quantitative assay to detect ABO blood group antibodies by flow cytometry in an isotype-specific manner (15). The anti-A/B IgG and IgM MFI histograms of a representative CVID patient and a representative healthy control are given in Figures 3A–D, where the *X*-axis represents the MFI given on a logarithmic scale and the *Y* -axis the cell number on a linear numeric scale. The filled histograms represent binding of an anti-A/B-negative control sample (AB serum), the bold lines (CVID) and hatched histograms (healthy control) represent the binding of IgM/IgG antibodies present in the tested serum that react with human red blood cells (RBCs). A statistically significant impairment in anti-A/B IgG formation was detected in CVID patients as compared to healthy controls (Figures 3A,B,E), while anti-A/B IgM-antibody titers were comparable between CVID patients and controls (Figures 3D,F).

### CVID patients produce no blood group anti-A/B IgG as detected by surface plasmon resonance using the Biacore^®^ device

To further discriminate between IgM and IgG anti-A/B response we added anti-IgM and anti-IgG antibodies to the SPR assay as described in the Section “Materials and Methods.” An additional binding of the anti-IgM and anti-IgG antibodies to the respective anti-A/B antibodies present in the test sample caused a measurable rise in the RU value that was proportional to the amount of IgM/IgG-anti-A/B antibodies present in the sample. Commercially available anti-blood group A/B mAbs in a concentration of 50 μg of anti-A IgM mAbs gave 2500 RU in this assay, while the limit of detection was observed at 300 ng of anti-A IgM, and 40 μg of anti-B IgM mAbs gave a maximum 1000 RU, while the limit of detection in this case was observed at 640 ng. When the assay was calibrated with 10 μg of anti-A IgG mAbs a maximum of 15,000 RU were measured, while the limit of detection for anti-A IgG was observed at 100 ng.

**Figure 4:**
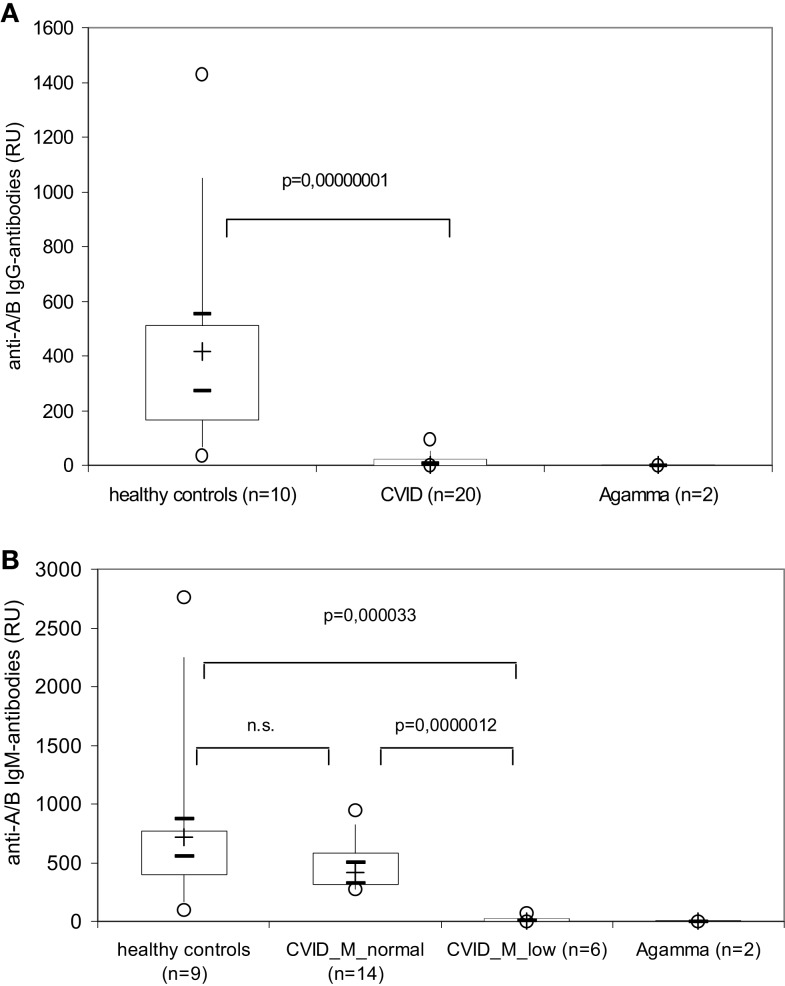
**Isotype-specific measurement of anti-A/B antibodies by surface plasmon resonance using the Biacore^®^ device in healthy controls, patients with CVID and XLA patients (Agamma)**. The amount of IgG- (upper panel) and IgM- (lower panel) anti-A/B antibodies present in the serum sample are depicted as resonance units (RU) using box plot diagrams, the median of the study group is represented by a cross, the interquartile range (IQR) is represented by the box, 5- and 95-percentile values are represented by the whiskers, and minimum and maximum values are represented by circles. The 95% confidence interval for the median calculated according to McGill et al. (21) is indicated by horizontal bars. The non-parametric Mann–Whitney *U*-test (two-sided *P* value) was used for statistical comparison between patients and healthy controls. CVID_M_low, CVID patients with very low to undetectable anti-A/B IgM-antibodies; CVID_M_normal, CVID patients with anti-A/B IgM-antibody titers that were comparable to the healthy control group.

While ≥90% of the healthy individuals presented with easily detectable (≥100 RU) anti-A/B IgM and IgG antibodies, anti-A/B IgG antibodies were undetectable or very low (<100 RU) in all 20 CVID patients investigated (Figure 4, upper panel), Six of these 20 patients also had very low to undetectable anti-A/B IgM-antibodies (CVID_M_low in Figure 4, lower panel), while levels of anti-A/B IgM-antibodies in the remaining patients (CVID_M_normal in Figure 4, lower panel) were comparable to the healthy control group.

### Affinity of blood group anti-A/B IgM antibody binding to blood group A/B trisaccharide antigens is not altered in CVID patients

The erythrocyte aggregation assay based on the Diamed-ID Micro Typing System as well as the flow cytometric method to quantify binding of anti-A/B antibodies to fixed human A or B red blood cells (ABO-FACS) cannot rank anti-A/B antibodies according to their strength of reaction with the corresponding blood group A/B trisaccharide antigens. In order to compare affinity of the anti-A/B IgM antibodies, we depleted serum IgG from healthy individuals with high blood group anti-A or B isoagglutinins with protein A- and protein G-affinity columns and measured the on and off rates of the anti-AB IgM antibodies to the respective trisaccharide immobilized on the CM-5 chip. For these experiments, amine-coupling of the trisaccharides to the surface of the CM5 sensor chip was used in order to guarantee physiological condition of binding (16–18). We found no difference in association (*k*_a_) and dissociation rate (*k*_d_) between CVID patients and controls depleted of IgG when sera were diluted to match with 700 RU (data not shown).

### Impaired blood group anti-A/B antibody response in CVID patients is associated with defective IgM-antibody response to pneumococcal polysaccharides

**Figure 5:**
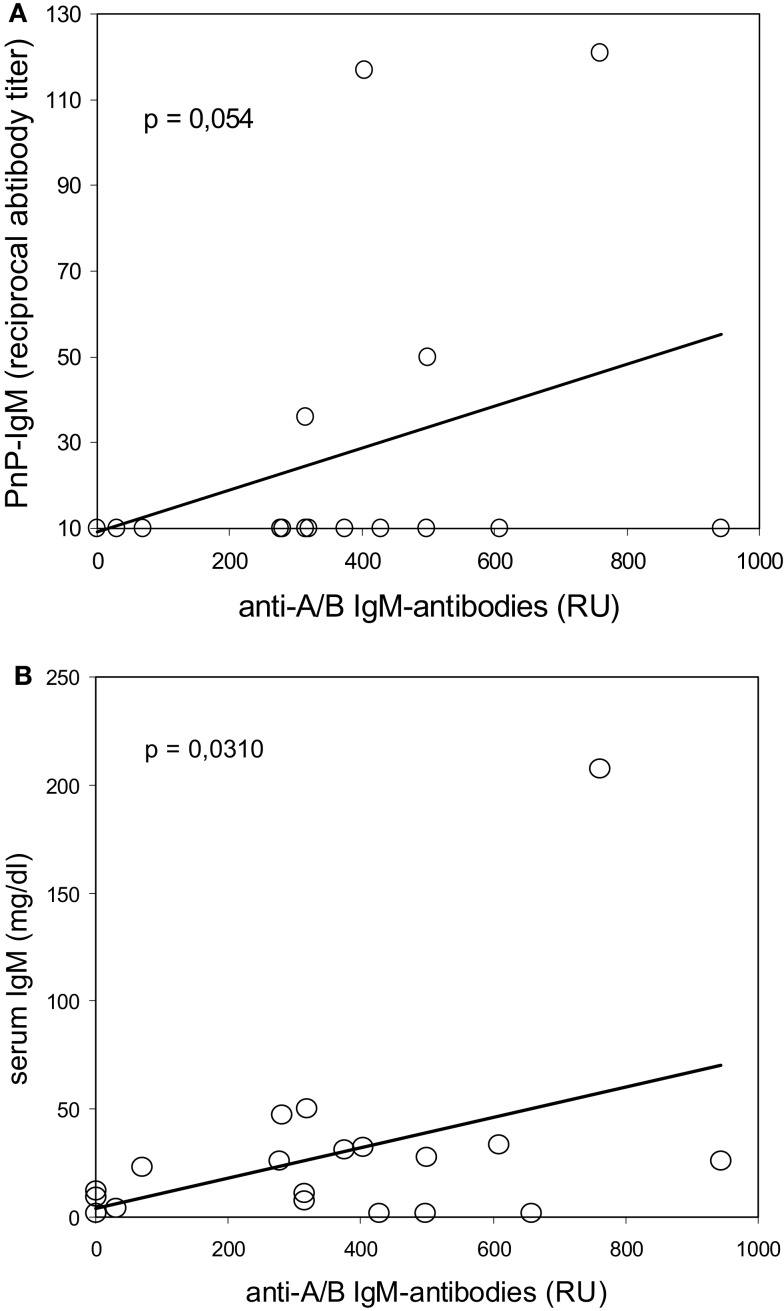
**Correlation between reduced blood group anti-A/B IgM antibodies and defective IgM-antibody response to pneumococcal polysaccharides (upper panel) or reduced serum IgM levels (lower panel) in CVID patients**. Serum IgM antibodies against 23-valent pneumococcal polysaccharides (PnP–IgM) were determined by ELISA, serum blood group anti-A/B IgM antibodies were assessed using isotype-specific surface plasmon resonance technology, serum IgM levels were measured by nephelometry. Results are depicted as reciprocal serum titer (for PnP–IgM), resonance units (RU, for anti-A/B IgM-antibodies), and mg/dl (for serum IgM). The Bravais–Pearson-correlation coefficient was calculated to determine a statistically significant correlation at a level of*P* < 0.05.

Antibodies against 23-valent PnP were determined by ELISA in 32 of the 34 CVID patients in order to find a correlation between the immune response to PnPs and blood group ABO antigens, because ABO- and PnP-antigens share similar carbohydrate moieties (22–24). Interestingly, among the 32 CVID patients in whom both PnP antibodies and isoagglutinins could be tested, nine patients showed no detectable isoagglutinins and no or only very low blood group anti-A/B antibodies in the SPR assay and also showed no PnP-specific IgM-antibody response. Among the remaining 23 CVID patients with detectable blood group anti-A/B isoagglutinins, only 15 showed no anti-PnP IgM-antibodies (*P* = 0.042, Chi-squared analysis). In addition, there was a trend toward a positive correlation between the levels of the patients’ anti-A/B IgM-response as measured using SPR and their serum anti-PnP IgM, and low levels of total serum IgM were significantly correlated with low levels of anti-A/B IgM-antibodies (Figure 5). Typical for all CVID patients was the incapability to produce PnP-specific IgG (Table 1), which was in accordance with a defective IgG-response to blood group ABO antigens.

## Discussion

Common variable immunodeficiency (CVID) comprises a heterogeneous group of patients with a multitude of immunological abnormalities that have in common a severely impaired antibody production with altered isotype-switch and defective affinity maturation of the antibodies produced (5). Dependent on the underlying defect, B cell activation and development might be compromised at different levels enabling B cells of certain patients with CVID to mount an IgM antibody response, while B cells of other CVID patients with more severe defects cannot produce any antibodies at all (25). Due to the profound antibody deficiency, the patients suffer from severe susceptibility to infections, and the demonstration of defective IgG antibody production provides the indication for immunoglobulin replacement therapy (26), as was proposed by an IUIS expert committee already many years ago (5). The present study investigated the capability of CVID patients’ B cells to respond to blood group ABO antigens and shows that 100% of the patients that receive long-term immunoglobulin replacement therapy based on their inability to produce IgG antibodies following vaccination or infection (26) also have a defect in IgG antibody formation against ABO blood group antigens, thus providing an additional diagnostic parameter of possible clinical-therapeutic relevance.

Anti-ABO antibodies are thought to be natural antibodies produced by all individuals with a functional immune system (14). Their immunologically active epitopes share carbohydrate moieties and appear first in infants at the age of 3–6 months when the developing immune system can react with microorganisms and environmental antigens that have similar carbohydrate epitopes as the blood group ABO antigens (22–24). Certain debate exists, however, whether anti-AB antibodies completely fulfill the criteria of natural antibodies (27). Natural antibodies were described to be encoded by germline variable (V) genes, show no somatic mutations and are produced predominantly by CD5^+^ B1 cells, at least in the mouse, although certain splenic CD5^−^ B2 cells were also capable of producing natural antibodies (28, 29). Natural antibodies are mostly of the IgM isotype, are therefore polyreactive and show a wide range of binding avidities (14, 30). High resolution crystallography has shown that anti-carbohydrate antibodies in germline configuration can recognize a range of structurally related carbohydrate epitopes (22). A repertoire of pre-existing ‘natural antibodies’ might exist in healthy individuals in the absence of immune stimulation which is encoded by a relatively small number of germline progenitors recognizing carbohydrate moiety with a define shape (31), although a second stage of selection for antibodies directed against blood group ABO antigens with higher affinity cannot be excluded at present.

Natural antibody formation has up to now not been studied in greater detail in patients diagnosed with CVID. This study shows that a defective IgG antibody response to protein and polysaccharide antigens that is characteristic for patients with CVID (26) is accompanied by a defect in the formation of natural IgG antibodies such as anti-A/B IgG-antibodies. Of the 34 patients investigated, we found only nine CVID patients with no detectable anti-A/B antibodies at all, similar to the eight patients with XLA that had no detectable peripheral B cells due to btk-deficiency. The remaining 25 patients showed detectable anti-A/B antibodies that were of the IgM isotype. In contrast, healthy individuals showed both anti-A/B IgM and IgG responses. These results are in accordance with previous studies showing defects in class switching in CVID patients (25, 26, 32). Interestingly, the association rate (*k*_a_) and dissociation rate (*k*_d_) of blood group specific anti-A/B IgM antibodies binding to defined blood group A/B trisaccharides was normal in CVID patients, indicating that the majority of CVID patients might produce blood group specific IgM antibodies that are functional intact.

The defective IgM response against A/B blood group antigens observed in CVID patients was accompanied by defective IgM antibody formation against pneumococcal capsular polysaccharides as well. One reason for the failure of CVID B cells to respond to pneumococcal and blood group polysaccharides could be insufficient triggering of TLRs and their signaling pathways, as TLR-activation is critical for T-independent antibody responses (33). CVID B cells were shown to have impaired responses to TLR7 and TLR9 agonists including poor cell proliferation, loss of cytokine production, and failure to produce and secrete IgG and IgA (34, 35). IFN-α could restore TLR7- and TLR9-triggered functional responses in B cells of CVID subjects with >0.5% peripheral isotype-switched memory B cells (34). An alternative explanation for the observed association between anti-A/B and pneumococcal IgM-antibodies could be a general decrease in IgM production as is reflected by the low level of total serum IgM in most CVID patients.

Patients with predominantly antibody deficiency who have a long history of clinical disease, in particular recurrent infections of the lower respiratory tract, are well known to be prone to developing organ damage such as chronic lung disease, which determines their long-term prognosis (36). Increased awareness for PID has made earlier diagnosis and initiation of adequate therapy in PID patients feasible, so that more and more patients with predominantly antibody deficiency lack a long history of clinical disease, making it necessary to initiate immunoglobulin replacement therapy based on laboratory findings rather than patient history. Detection of impaired IgG antibody formation has been suggested as a laboratory parameter to facilitate the decision on early immunoglobulin replacement in hypogammaglobulinemic patients (26). Currently used diagnostic criteria for CVID, e.g., the criteria proposed by an IUIS expert committee (5), the most commonly used European Society for Immunodeficiencies/Pan American Group for Immunodeficiency (ESID/PAGID) definition of CVID (3) or the 2014 diagnostic criteria for the ESID registry (4) propose defective isoagglutinins and impaired antibody formation after vaccination or infection as equivalent criteria. SPR analysis proved to be the most specific and sensitive method for the detection of anti-blood group A/B IgG antibodies and applying this method a pronounced defect in IgG antibody production could be demonstrated and defined in all CVID patients tested. As can be seen in Figure 1, conventional methodology such as the erythrocyte aggregation assay based on the Diamed-ID Micro Typing System was unable to detect defective antibody production in the majority of CVID patients, as a considerable proportion (approximately 2/3) of these patients can produce normal levels of anti-A/B IgM antibodies. Furthermore, SPR analysis of anti-A/B antibodies can be applied to ascertain early diagnosis of IgG antibody deficiency in CVID patients, while at present, antibody failure has to be proven by the lack of response to vaccination. The clinical application of SPR analysis for the diagnosis of antibody deficiency certainly requires further development. Comparable efforts have been undertaken for the application of this method in the rapid quantitation of blood group antibodies (16), and more recently a 96-well-plate format has been presented for clinical applications of SPR in serum antibody quantification (37). In contrast to microtyping, both flow cytometry and SPR can detect anti-A/B antibodies in an isotype-specific manner. In addition to a greater sensitivity in detecting anti-A/B antibodies, further advantages of the SPR method include the use of molecularly well defined, standardized blood group antigens as compared to the interindividual variations in donor erythrocytes used as the particulate antigen in flow cytometry, and the ability to detect biologically relevant antibody specificities, as the carbohydrate antigens are presented in a physiological manner.

## Author Contributions

HW and MF were the principal investigators, provided laboratory resources, analyzed clinical and immunological data, wrote the first manuscript draft together, critically participated in all revisions of the manuscript and take primary responsibilities for the paper. WW performed all isoagglutinin and SPR analyses and analyzed the results, MW and GB performed the ABO FACS measurements and analyzed the results, ME provided clinical patient data and laboratory resources, participated in data analysis and interpretation and critically reviewed the initial draft and all revisions of the manuscript, CB participated in data interpretation.

## Conflict of Interest Statement

The authors declare that the research was conducted in the absence of any commercial or financial relationships that could be construed as a potential conflict of interest.
